# Impaired airway epithelial miR-155/BACH1/NRF2 axis and hypoxia gene expression during RSV infection in children with down syndrome

**DOI:** 10.3389/fped.2025.1553571

**Published:** 2025-05-21

**Authors:** Allison Welham, Elizabeth Chorvinsky, Surajit Bhattacharya, Kyle Salka, Betelehem Solomon Bera, Woudasie Admasu, Maria C. Straker, Maria J. Gutierrez, Jyoti K. Jaiswal, Gustavo Nino

**Affiliations:** ^1^ Division of Pediatric Pulmonary and Sleep Medicine, Children’s National Hospital, Washington, DC, United States; ^2^ Center for Genetic Medicine Research, Children’s National Research Institute, Washington, DC, United States; ^3^ Department of Pediatrics, School of Medicine and Health Sciences, George Washington University, Washington, DC, United States; ^4^ Jacobs School of Medicine and Biomedical Sciences, University of Buffalo, Buffalo, NY, United States; ^5^ Division of Pediatric Allergy, Immunology and Rheumatology, Johns Hopkins University, Baltimore, MD, United States

**Keywords:** trisomy 21 (Down syndrome), RSV (respiratory syncytial virus), airway epithelia, miR-155, hypoxia

## Abstract

**Background:**

Children with Down Syndrome (DS) are at high risk for severe respiratory syncytial virus (RSV) infections. DS is associated with impaired cellular responses to oxidative stress and hypoxia; however, these abnormalities have not been explored in trisomy 21 (TS21) airway epithelial cells (AECs) during RSV infection. Understanding these defects is key to identifying factors contributing to severe RSV infections in this high-risk group.

**Methods:**

AECs from children with and without DS were analyzed at baseline and after RSV infection to assess NRF2-induced protective genes against oxidative stress and hypoxia, including the enzyme heme oxygenase 1 (HO-1). To investigate DS-specific defects, we focused on miR-155 and BACH1, which regulate NRF2 signaling and HO-1 expression, and are both encoded on chromosome 21. RNA-seq analyses were performed to examine genome-wide hypoxia-related gene responses in control and TS21 AECs at baseline and after RSV infection.

**Results:**

Our findings show that miR-155 inhibits BACH1, leading to increased NRF2-driven HO-1 expression in euploid AECs. In contrast, TS21 AECs from children with DS exhibited impaired HO-1 induction following miR-155 treatment. This was attributed to reduced transcription of the HMOX1 gene, which encodes HO-1, along with global downregulation of hypoxia response genes in DS at baseline and after RSV infection in TS21 AECs.

**Conclusions:**

Severe RSV infections in children with DS may be linked to intrinsic defects in AEC responses to hypoxia, including NRF2-driven cytoprotective enzymes like HO-1. These findings offer new mechanistic insights into RSV pathophysiology and potential therapeutic targets in children with DS.

## Introduction

Respiratory syncytial virus (RSV) is a leading cause of hospitalization and mortality in young children (1, 2). However, the mechanisms governing protective airway responses against RSV, particularly in high-risk populations like Down syndrome (DS), are not well understood (3, 4). A meta-analysis of over a million children found that those with DS have a nine-fold higher risk of RSV-related hospitalization and death (3). Compared to euploid children, those with DS experience more severe respiratory outcomes, including longer hospital stays and greater need for mechanical ventilation during severe RSV infections (3). Despite this, abnormalities in the airway protective response to RSV in children with DS remain largely unexplored.

It is well known that severe RSV infections in young children cause oxidative stress and hypoxic injury in airway cells and tissues (5–7). Airway cytoprotective responses to RSV are driven by the nuclear factor erythroid 2-related factor 2 (NRF2) (5–7), which activates the expression of antioxidative enzymes, such as heme oxygenase 1 (HO-1) (8, 9). Reduced NRF2 responses in the airways are linked to more severe respiratory disease in infants with viral bronchiolitis and in experimental RSV models (5–7). Notably, while DS is associated with impaired responses to oxidative stress and hypoxia in several cell systems (10–12), these abnormalities have not been investigated in trisomy 21 (TS21) cells in the context of RSV infection.

TS21 presents a unique model for studying dysregulated NRF2 responses during RSV infection because BACH1, the canonical inhibitor of NRF2, is encoded on chromosome 21 (Hsa21) (12–14). Additionally, miR-155, which targets BACH1, is also located on Hsa21, leading to dysregulated expression of both miR-155 and BACH1 in TS21 cells (10–12, 15). Our team identified that miR-155 is consistently induced in the airways of infants with viral bronchiolitis, where it regulates inflammatory and antiviral responses, including interferon (IFN) signaling (16, 17). Interestingly, TS21 cells also exhibit heightened IFN signaling due to the triplication of four IFN receptor genes on Hsa21 (18–20). Together, these findings suggest that, despite increased IFN activity, other host protective mechanisms, such as the miR-155/BACH1/NRF2 axis, may be impaired in TS21 cells during RSV infection.

In this study, we used airway epithelial cells (AECs), the primary point of entry and host defense against RSV (21), to investigate the cooperative roles of miR-155, BACH1, and NRF2-driven cytoprotective responses in euploid children and those with DS. We hypothesized that in AECs from euploid children, miR-155 regulates the BACH1/NRF2 axis, leading to increased HO-1 expression. In contrast, in children with DS, intrinsic defects in TS21 AECs impair hypoxia gene responses, including reduced NRF2-driven antioxidative enzymes like HO-1, both at baseline and during RSV infection. Our findings indicate that severe RSV infections in children with DS may be linked to altered airway epithelial cytoprotective responses to oxidative stress and hypoxic injury. These results provide new mechanistic insights and potential therapeutic targets to mitigate the severe impact of RSV infections in children with DS.

## Methods

### Study participants

A total of 13 infants and children born at term (ages 0.9–13 years) with and without DS were enrolled for our studies. This study was approved by the Institutional Review Board (IRB) at Children’s National Hospital (CNH) (IRB Pro00012323, Pro00003441, STUDY00000511) and included parental written informed consent. All patient data were de-identified. Donor characteristics are detailed in Table E1.

### AEC sample collection and cultures

Human nasal AECs were collected via brushing as described (22). Collected AECs were established and cultured using previously published methods (23). AECs from both euploid and DS donors were plated on collagen coated 6 well dishes (pureCol) at a density of 90,000–150,000 cells/well for approximately 24 h in conditional reprogramming cell (CRC) media (Promocell) (23). Plated AECs were treated with siRNA non-targeting control (Thermo Fisher) or miRVana microRNA-155 mimic (Thermo Fisher) in Opti-MEM media (Gibco) for 6.5 h, at which point media was changed to CRC media. 24 h post transfection, AECs were exposed to viral mimic (Poly I:C 10 ng/µl) or 72 h post transfection, AECs were infected with diluted RSV virus stock in RA only media (Promocell) to an MOI of 1. RNA and protein lysates were collected using TRIzol reagent (Thermo Fisher) and RIPA Buffer (Thermo Fisher) 24 h after exposure to virus or viral mimic. Protein expression was quantified using Western blotting. Cellular and molecular studies are detailed in the Supplementary Methods.

### RNAseq and affymetrix methods

Total RNA was isolated using phase separation via the phenol-chloroform method and RNA quality was assessed using the Agilent 2100 Bioanalyzer. Bulk RNA sequencing data was analyzed using methods previously stated (22). Reads were aligned to the human reference (hg38), and the counts were estimated with RSEM (version1.3.1). Low read counts were filtered out (<200 reads), then normalization and differential expression were performed with DESeq2 (version1.38.3) using an adjusted *p*-value threshold of <0.05 and an average log2 fold change of >1. Microarray was performed and data analyzed by methods previously described (24). Following the Affymetrix protocol the fragmented cDNA is labeled and hybridized to Clariom S Human microarrays (4.0), followed by scanning using Affymetrix Gene Chip scanner. The raw CEL files were then quality filtered, normalized and visualized using Transcriptomic Analysis Console (TAC; Affymetrix), version 4.0. RNA sequencing and statistical analysis are detailed in the Supplementary Methods.

## Results

### Target screening of miR-155 effects in human infant airway epithelial cells at baseline and following activation of antiviral response

**Figure 1:**
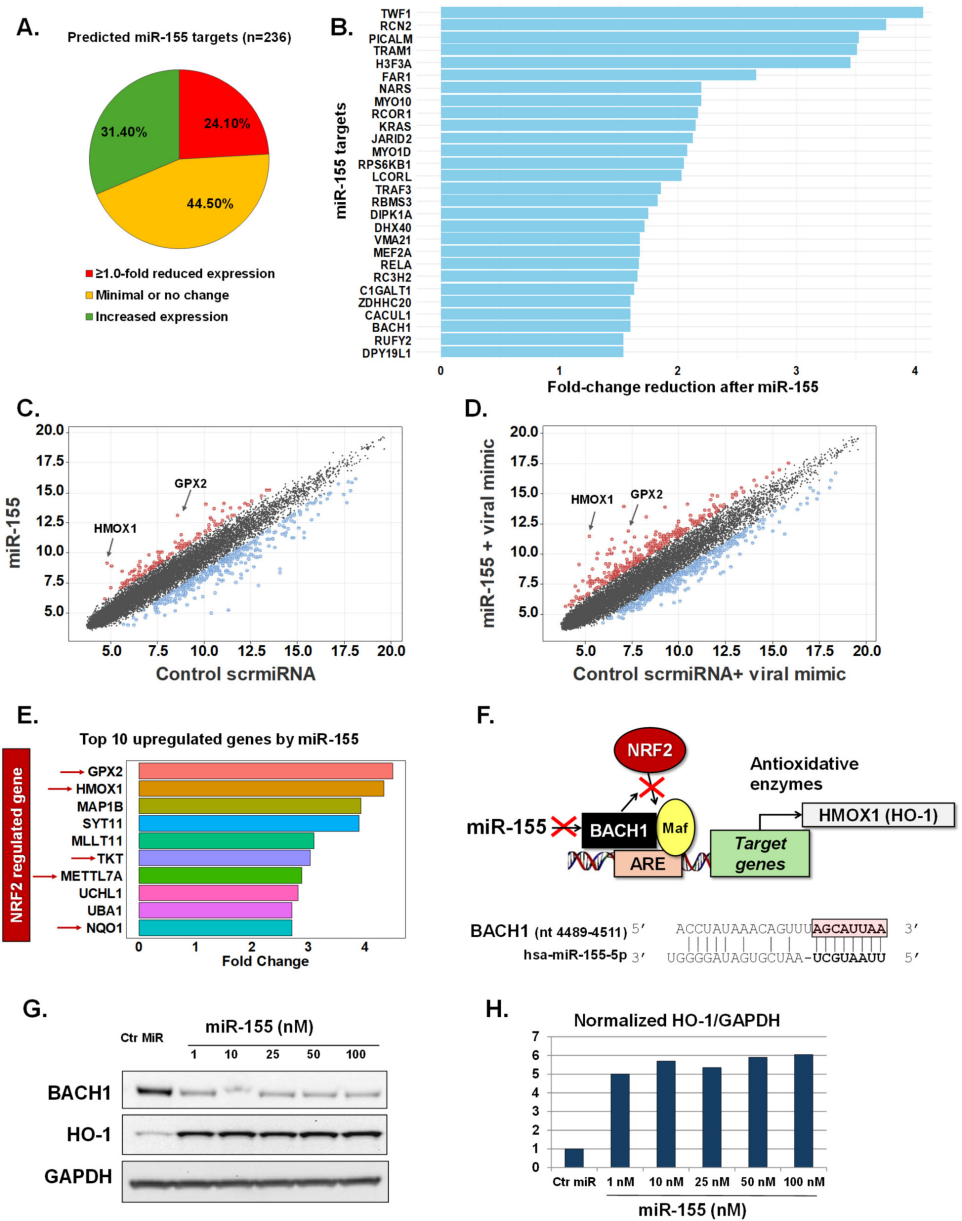
miR-155 effects in human infant airway epithelial cells at baseline and following activation of antiviral response. Expression changes of predicted miR-155 target genes (*n* = 236) after transfection to miR155 mimic **(A)**. Bars represent miR155 target genes with the highest reduced fold change after miR155 transfection (*n* = 28) **(B)**. Scatter plots show fold changes of all genes after treatment with control scramble miRNA sequence (scrmiRNA) vs. treatment with miR155 mimic at baseline **(C)** and after treatment with the viral mimic poly I:C **(D)**. Top 10 genes upregulated after treatment with miR155 mimic including 5 genes in the NRF2 pathway **(E)**. Schematic of miR155’s regulatory role in the NRF2 pathway and complementary sequence to BACH1 **(F)**. Immunoblotting showing dose-response changes in BACH1 and HO-1 protein levels after miR155 treatment **(G,H)**.

Utilizing transcriptomic arrays we evaluated genome-wide changes induced by miR-155 transfection in pediatric nasal AECs. To minimize variability, primary AECs from a single 10-month-old euploid infant donor were utilized. To control for potential off-target effects from miRNA transfection, we used a non-targeting scramble miRNA sequence (scrmiRNA) as a control. The top predictive targets of miR-155, along with their corresponding scores and rankings, were defined using a miRNA prediction and functional annotation database (25, 26). This analysis identified 236 genes with miR-155 target scores of ≥80 (Supplementary Table E2). Following miR-155 transfection, 57 of these targets (24.1%) showed a ≥1.0-fold reduction in expression. In contrast, 105 targets (44.5%) showed minimal or no change, while 74 targets (31.3%) had increased expression (Figure 1A). Only 28 miR-155 targets demonstrated ≥1.5-fold inhibition in human infant AECs after miR-155 transfection (Figure 1B).

Given that the effects of miR-155 on predicted targets were relatively modest in human infant AECs, we expanded our screening to include all genes. We identified 333 genes with ≥1.5-fold reduced expression after miR-155 exposure (Figure 1C, Supplementary Table E3). Notably, 129 genes also exhibited ≥1.5-fold increased expression relative to the control scrmiRNA (Figure 1C, Supplementary Table E3). Two genes, GPX2 and HMOX1, demonstrated the most striking pattern with >4-fold upregulation after miR-155 transfection (Figure 1C, Supplementary Table E3). To evaluate the robustness of these findings and their relevance to antiviral responses, we replicated our experiments, exposing the pediatric AECs to a synthetic viral mimic (dsRNA, poly I:C, 10 ng/μl for 24 h.). As expected, the viral mimic induced widespread transcriptomic changes, primarily characterized by the upregulation of IFN-inducible antiviral genes (Supplementary Table E4). Consistent with our initial results, in the context of antiviral responses, miR-155 upregulated GPX2 expression (4.6-fold change, Figure 1D, Supplementary Table E5). Furthermore, we found that the change in HMOX1 expression was even greater than what was observed under resting conditions (6.28-fold change after poly I:C, Figure 1D, Supplementary Table E5).

#### Human infant airway epithelial miR-155-driven regulation of the BACH1/NRF2 axis

Pediatric AECs had a marked upregulation of HMOX1 and GPX2 after exposure to miR-155, both at baseline and following viral mimic stimulation (Figures 1C,D). These genes encode the antioxidant enzymes heme oxygenase-1 (HO-1) and glutathione peroxidase 2 (GPX2), both of which are induced by master antioxidant transcription factor NRF2 (12–14). Interestingly, we found that five of the top 10 genes upregulated by miR-155 are also known to be induced by NRF2, including NAD(P)H Quinone Dehydrogenase 1 (NQO1), methyltransferase like 7A (METTL7A) and Transketolase (TKT) (27–30) (Figure 1E). These findings suggested a potential link between miR-155 and the activation of NRF2-driven genes in human infant AECs. To explore this further, we focused on BACH1, the canonical inhibitor of NRF2 signaling (12–14), and also a predicted target of miR-155 (25, 26). Sequence analysis revealed that the BACH1 gene contains a specific binding site for miR-155 in its 3′-untranslated region (3′UTR) (Figure 1F), making it a high-ranking predicted target (Rank #3, score 99, Supplementary Table E2).To evaluate the effect of miR-155 on BACH1/NRF2 signaling, we assessed the protein expression of HO-1 (encoded by HMOX1), a well-established marker of NRF2 activation and a cytoprotective enzyme involved in redox and hypoxic stress (8, 9). Western blotting revealed a dose-dependent reduction in BACH1 and a corresponding increase in HO-1 levels following miR-155 transfection (Figures 1G,H). Collectively, our results indicate that miR-155 can modulate antioxidative responses in pediatric AECs by downregulating BACH1, thereby promoting NRF2-driven activation of cytoprotective enzymes, such as HO-1.

#### Trisomy 21 alters airway epithelial miR-155-driven regulation of the BACH1/NRF2 axis

**Figure 2:**
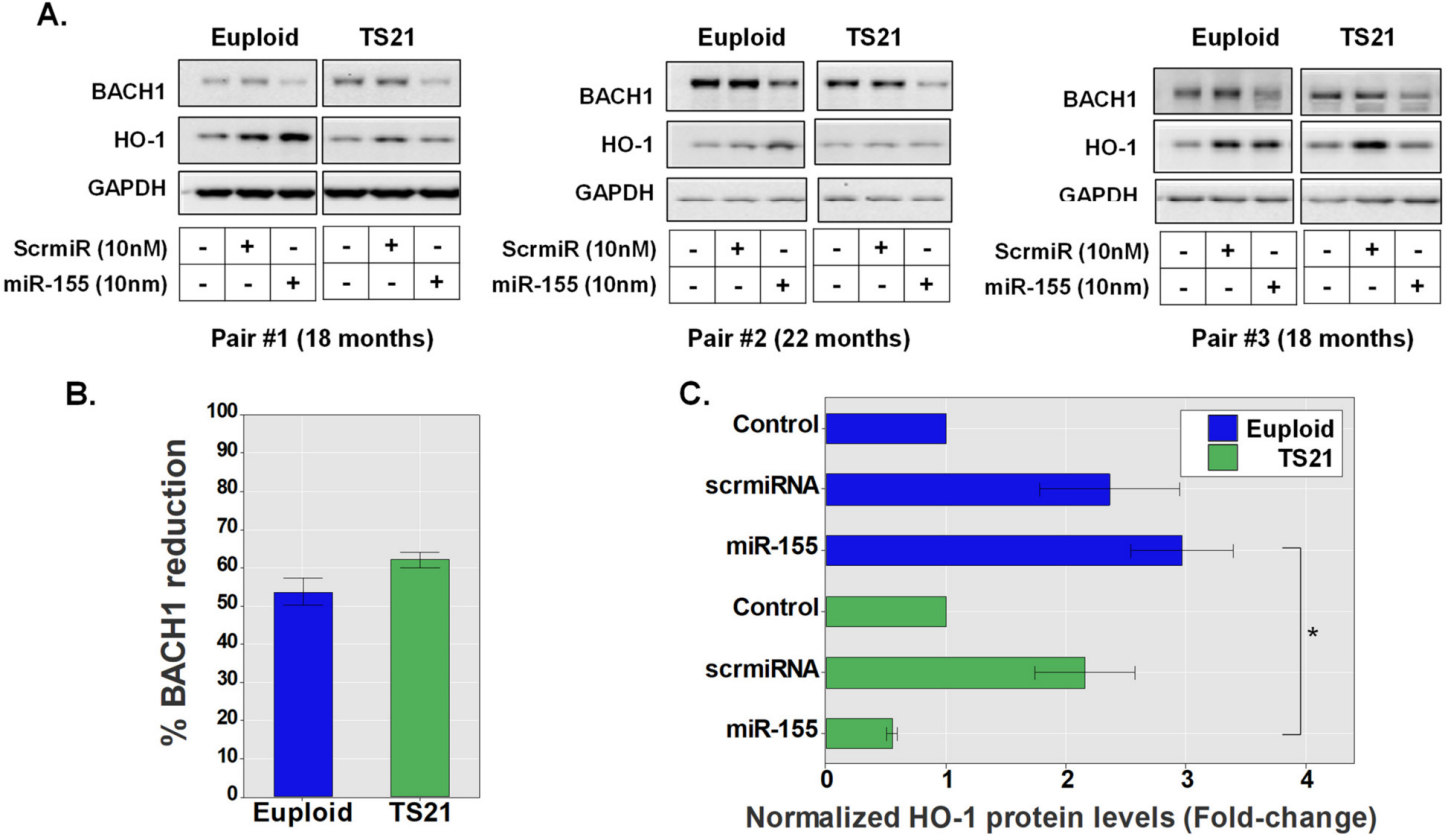
Trisomy 21 alters airway epithelial miR-155-driven regulation of the BACH1/NRF2 axis. Immunoblotting of BACH1 and HO-1 protein levels after treatment with a control scramble miRNA sequence (scrmiRNA) or miR155 mimic in TS21 AECs (*n* = 3) and age-matched euploid controls (*n* = 3) **(A)**. Bars represent the percentage of BACH1 normalized protein level reduction by miR155 mimic in TS21 and euploid AECs **(B)**. Normalized HO-1 protein levels in transfected AECs of both TS21 and euploid donors (C), *p < 0.05.

Our experiments demonstrate that miR-155 increases HO-1-mediated responses by inhibiting BACH1 in pediatric AECs (Figure 1). Interestingly, BACH1 and miR-155 are both encoded in the hsa21 resulting in triplication of these genes in TS21 cells (10–12, 15). The latter of which has been linked to alterations within the BACH1/HO-1 axis as well as miR-155 expression in DS in other cell systems (10–12). Thus, we next examined whether AECs from children with DS also demonstrate altered miR-155-mediated regulation of the BACH1/HO-1 axis. To compare BACH 1 and HO-1 protein levels following miR-155 transfection in AECs from three age-matched pediatric pairs of DS (TS21, *n* = 3) and euploid controls (*n* = 3), we utilized immunoblotting (Figure 2A). These experiments showed that miR-155 transfection resulted in a reduction of over 50% in BACH1 protein levels in both groups, trending toward a more prominent effect in children with DS (56.2% reduction in the euploid group, 62% reduction in the DS group, *p* = 0.1, Figure 2B). In pediatric euploid AECs, miR-155 transfection induced a 3-fold increase in HO-1 protein levels relative to no treatment (Figure 2C), while a more modest increase in HO-1 was also observed following scrmiRNA exposure in both groups (Figure 2C). Notably, in pediatric TS21 AECs, despite significant reduction in BACH1 (Figure 2B), miR-155 failed to increase HO-1 levels in any of the DS donors, in contrast to the prominent effect seen in euploid AECs (Figure 2C).

#### Human airway epithelial cell expression of hypoxia gene responses is impaired by trisomy 21

**Figure 3:**
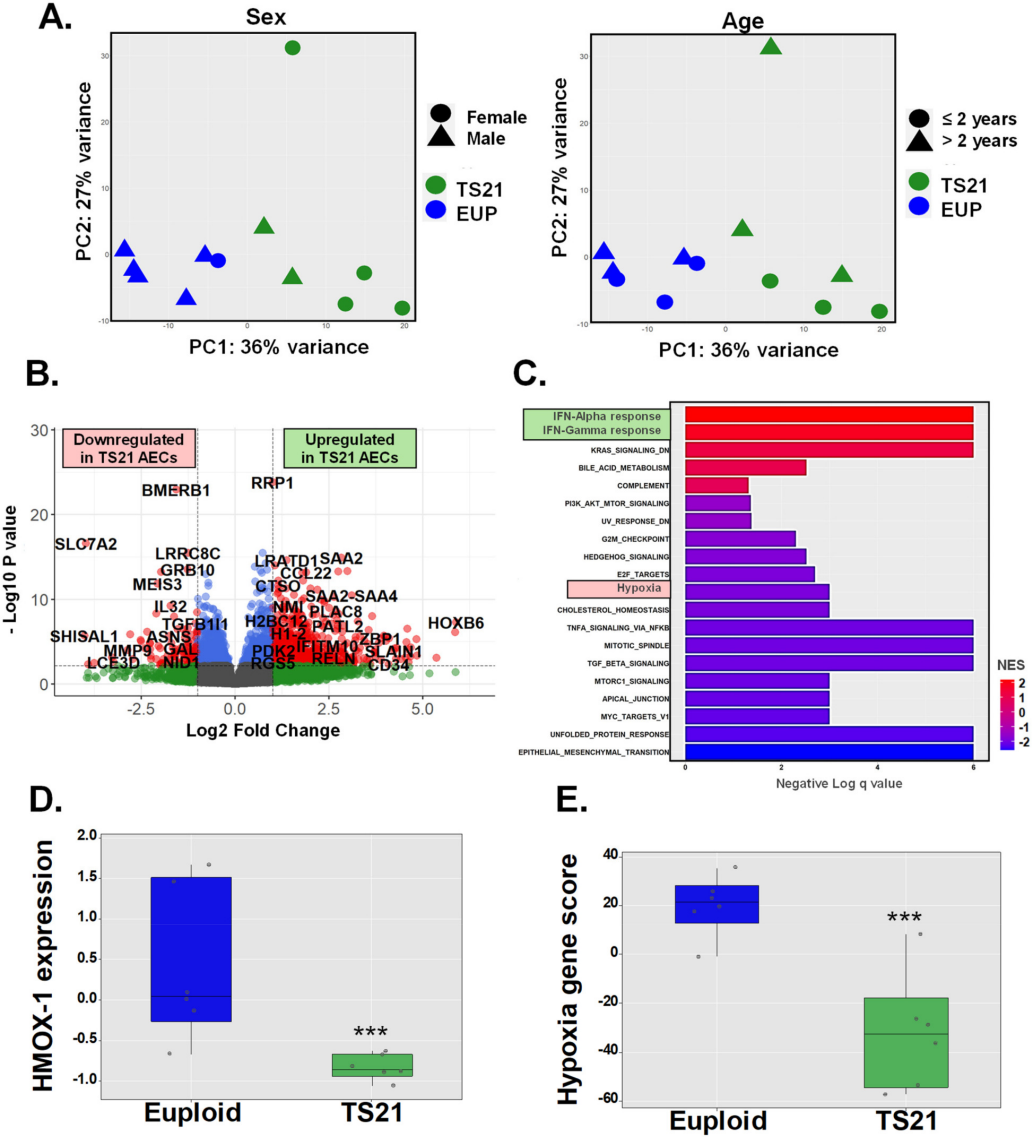
Human airway epithelial cell expression of hypoxia gene responses is impaired by trisomy 21. Principal component analysis (PCA) shows sex independent and age independent clustering of euploid and TS21 samples at baseline **(A)**. Volcano plot of differentially expressed genes in euploid vs. TS21 AECs at baseline **(B)**. Gene ontology (GO) analysis showing pathways of differentially expressed genes between euploid vs. TS21 AECs **(C)**. Normalized HMOX-1 expression **(D)** and average of hallmark hypoxia gene *z*-scores **(E)** in euploid vs. TS21 AECS at baseline, ***p < 0.001.

Prior studies have linked DS to widespread alterations in cellular responses to oxidative stress and hypoxia, including the downregulation of HMOX1 (10–12.) Based on this, we hypothesized that the inability of miR-155 to induce HO-1 in AECs from children with DS results from an intrinsic defect in HMOX1 transcription, which may be part of a broader disruption in the induction of genetic responses to hypoxia. To test this hypothesis, we compared transcriptomic profiles (RNA-seq) in AECs from six pediatric donors including euploid and DS groups (Figure 3). Principal component analysis (PCA), adjusted for age and sex, revealed a clear separation between AECs from euploid children (*n* = 6) and those from children with DS (*n* = 6) (Figure 3A). We identified a total of 2171 differentially expressed genes (DEGs), with 962 downregulated and 1209 upregulated in TS21 AECs (Figure 3B). Gene ontology (GO) analysis using hallmark pathways of the Molecular Signatures Database (MSigDB) (31) revealed that the “*Hallmark Interferon Alpha Response*” and “*Hallmark Interferon Gamma Response*” pathways were the most upregulated in TS21 AECs, consistent with the known upregulation of IFN genes in DS (18–20, 32–35) (Figure 3C). In contrast, “*Hallmark Hypoxia*” gene responses were among the downregulated pathways in DS (Figure 3C). This Hallmark Hypoxia MSigDB pathway containing 200 genes (Supplementary Table E6) included HMOX1, which was significantly downregulated in AECs from children with DS compared to the euploid group (Figure 3D). Using a module score to integrate the 50 genes from the Hallmark Hypoxia MSigDB pathway expressed in human AECs (with >10 reads across samples, Supplementary Table E7), we identified a downregulation in the induction of genetic hypoxia responses in pediatric TS21 AECs (Figure 3E). These results demonstrate that the intrinsic defect in HMOX1 transcription in TS21 cells is part of a broader disruption in DS, characterized by reduced expression of airway epithelial genes involved in hypoxia responses.

### Children with Ds have impaired airway epithelial induction of hypoxia genes during RSV infection

**Figure 4:**
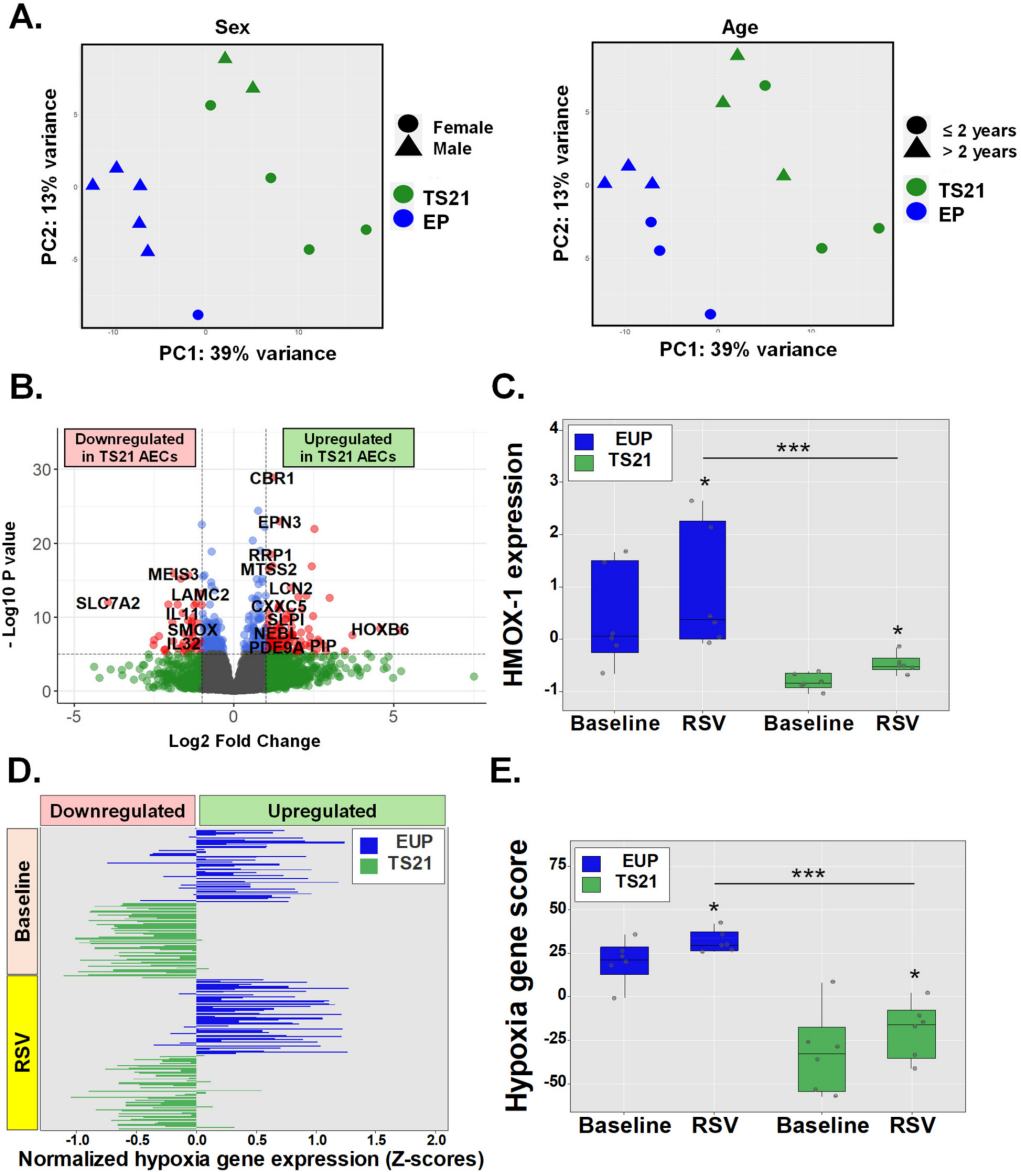
Children with DS have impaired airway epithelial induction of hypoxia genes during RSV infection. Principal component analysis (PCA) shows sex independent and age independent clustering of euploid and TS21 samples during RSV infection **(A)**. Volcano plot of differentially expressed genes in euploid vs. TS21 AECs during RSV infection **(B)**. Normalized HMOX-1 expression in euploids vs. TS21 AECs at baseline and during RSV infection **(C)**. Horizontal bars represent Z-scores of each hallmark hypoxia genes (*n* = 50) **(D)** and averaged hypoxia z-scores **(E)** in euploids vs. TS21 AECs at baseline and during RSV infection, *p < 0.05 ***p < 0.001.

Host AEC responses to RSV and other viral infections involve the induction of NRF2-driven cytoprotective molecules which help mitigate oxidative stress and hypoxic cell injury (5–7). Failure to induce these NRF2-mediated cytoprotective responses in the airways has been associated with severe RSV infections in human infants and animal models (5–7). While antiviral IFN pathways are upregulated in TS21 (18–20), children with DS remain at increased risk for severe RSV infections (3, 4), suggesting other host AEC responses to RSV infection may be compromised in these children. Given that TS21 AECs exhibit intrinsic AEC dysregulation of genes involved in hypoxia responses in DS (Figure 3), we hypothesized that children with DS would similarly show impaired activation of these cytoprotective responses to hypoxia during RSV infection. To test this, we compared RNA-seq data from AECs of euploid children (*n* = 6) and children with DS (*n* = 6) before and after RSV exposure. PCA adjusted for age and sex revealed a clear separation between AECs from euploid and DS children following RSV infection (Figure 4A). We identified 541 DEGs, with 147 being downregulated and 394 upregulated in TS21 AECs (Figure 4B). Notably, while HMOX1 was part of the RSV-induced transcriptional program in both groups, its expression was significantly reduced in TS21 AECs relative to euploid cells after RSV exposure (Figure 4C). Furthermore, when we integrated 50 hypoxia-related genes expressed in the hypoxia response module score (Table E6), we found that TS21 AECs had a significantly impaired induction of most hallmark hypoxia genes during RSV infection (Figure 4D) leading to an overall reduction in the hypoxia response module score in the DS group (Figure 4E).

## Discussion

Children with DS are at significantly higher risk for severe RSV infections (3, 4). While previous research has highlighted interferonopathy as a key contributor to the dysregulated immune response observed in DS (18–20), other protective responses during viral infection in TS21 cells have not been thoroughly explored. Specifically, DS is associated with widespread alterations in responses to oxidative stress and hypoxia, but these abnormalities have not been examined in TS21 AECs during RSV infections. This study aims to address this gap by investigating pathways associated with oxidative stress and hypoxic injury during RSV infection in pediatric AECs, focusing on miR-155 and BACH1—key regulators of NRF2 and hypoxia signaling pathways—both of which are encoded on Hsa21 (10–12, 15).

In this study, our initial screening in euploid AECs revealed that treatment with miR-155 increases the expression of NRF2-regulated genes. We focused on miR-155 because prior studies have consistently shown its induction during RSV infections (16, 17) and its critical role regulating airway inflammatory and antiviral responses (16, 36). The mechanisms by which miR-155 controls gene expression appear to be cell-specific due to context-dependent targeting and regulation (37–39). In circulating immune cells, miR-155 potentiates NF-κB activity and induces the production of pro-inflammatory cytokines via the suppression of SHIP1 and SOCS1, both of which are miR-155 targets (39). Our results show that miR-155 also plays an important role in AEC redox homeostasis by suppressing BACH1, the canonical inhibitor of NRF2 (12–14). This suppression leads to the activation of NRF2-driven enzymes, such as HO-1, in euploid AECs, but not in TS21 AECs.

The failure of miR-155 to activate NRF2-mediated responses in TS21 AECs may contribute to RSV bronchiolitis severity in DS. NRF2-driven enzymes play a critical role in the antioxidant and cytoprotective response to cellular stress signals and are key components of host defense during RSV infection (5–7). Studies in NRF2 knockout (NRF2-KO) mice have shown RSV infection results in increased airway obstruction and body weight loss, along with elevated levels of inflammatory cytokines (e.g., IL-1β, IL-10, IL-6, TNF-α) in the lungs compared to wild-type controls (5–7). Additionally, impairment of the NRF2 response led to higher viral replication and viral titers in these mice (5–7). Clinical studies have also linked reduced NRF2 signaling to worse patient outcomes during RSV infection (40). Children admitted to the pediatric intensive care unit (PICU) during RSV infections exhibited lower NRF2-driven antioxidant gene expression compared to non-PICU patients (40). When grouped by bronchiolitis severity, decreased NRF2-inducible gene expression was observed in children with the highest severity scores (40). The cytoprotective functions of NRF2 signaling during RSV infection are likely even more relevant for children with DS, who have approximately a nine-fold higher risk of RSV-related hospitalization and death (3).

Our results also revealed reduced HMOX1 expression, the gene encoding HO-1, both at baseline and during RSV infection in TS21 AECs. While HO-1 is one of many downstream enzymes involved in protecting against oxidative stress and hypoxia within the NRF2 pathway (8, 9), it also plays a specific role in reducing RSV-related lung pathogenesis. Studies have shown that pharmacological induction of HO-1 in mice reduces RSV replication and lung inflammation, as evidenced by decreased neutrophil infiltration in the airways and lower levels of cytokine and chemokine production (41). Moreover, in macrophages from HO-1 knockout (KO) mice, activation of Interferon Response Factor 3 (IRF3) is diminished, leading to impaired IFN-*β* production and reduced IRF3 phosphorylation during viral infection (42). Together, these findings underscore the critical role of HO-1 in the antiviral response to RSV infections. Impaired HO-1 responses in TS21 AECs may have multiple detrimental effects during RSV infections, including altered IFN responses, increased inflammatory infiltration of the airways, and reduced cytoprotection against oxidative stress and hypoxia.

In addition to reduced HMOX1 expression in TS21 AECs, our results revealed global impairment in hypoxia-related genes in AECs from DS donors. Hallmark hypoxia genes were obtained from the Molecular Signatures Database (MSigDB), which includes a cascade of molecules activated by hypoxia-inducible factors (HIFs) to adapt to oxygen deprivation (31). Hypoxia-related genes identified in AECs regulate key processes such angiogenesis (e.g., VEGF), glycolysis (e.g., HK1, ALDOC, PGM1), and tissue remodeling (e.g., TGFBI). The reduced constitutive expression of these hypoxia-related genes in TS21 AECs aligns with the widespread alterations observed in individuals with DS, which involve deficits in pathways regulating oxidative stress and hypoxic injury in other cell systems (10–12).

While hypoxia-related genes were upregulated during RSV infection in AECs from all children, they were significantly reduced both at baseline and during viral infection in AECs from DS donors compared to those from euploid children. These findings provide new insights into the pathogenesis of severe RSV infections in children with DS. During severe RSV bronchiolitis, viral replication leads to cell damage, triggers an inflammatory response, and disrupts oxygen distribution, contributing to local hypoxia and respiratory compromise (2, 43). Activation of HIF and hypoxia gene responses is essential for cellular adaptation, cytoprotection, and IFN-mediated antiviral responses that inhibit RSV infection (44). Therefore, the global impairment in hypoxia gene responses observed in AECs from children with DS may be a crucial factor contributing to the increased susceptibility to severe RSV infections in this vulnerable population.

A limitation of this study is the lack of data on miR-155 expression in TS21 AECs at baseline and in response to RSV infection. While its downstream targets are well-studied, the upstream regulators of miR-155, particularly in TS21, remain unexplored. Additionally, the small sample size, with all but one subject being male, could affect the findings, as sex differences in miR-155 expression may exist. In summary, our findings in AECs from children with and without DS, both at baseline and during RSV infection, revealed the following key points: (1) miR-155 inhibits BACH1 in pediatric AECs, leading to an increase in NRF2-driven HO-1 expression in euploid children. (2) TS21 impairs miR-155/BACH1 regulation, resulting in a lack of HO-1 induction in AECs from pediatric DS donors. (3) The defect in HO-1 induction in TS21 AECs is due to reduced transcription of HMOX1, the gene encoding HO-1, which is part of a broader downregulation of hypoxia-related genes in these cells. (4) Following RSV infection, AECs from children with DS show a significant reduction in virus-induced transcription of HMOX1, along with a global disruption in airway epithelial genes involved in hypoxia responses. Collectively, these results suggest that severe RSV infections in children with DS may be linked to intrinsic defects in airway epithelial responses to hypoxia, including impaired NRF2-driven antioxidative cytoprotective enzymes like HO-1. Interventions aimed at restoring these cytoprotective mechanisms in TS21 AECs may help reduce the burden of severe RSV infections in children with DS.

## Data Availability

The authors acknowledge that the data presented in this study must be deposited and made publicly available in an acceptable repository, prior to publication. Frontiers cannot accept a manuscript that does not adhere to our open data policies.
