# Supplementary material for: Impaired airway epithelial miR-155/BACH1/NRF2 axis and hypoxia gene expression during RSV infection in children with down syndrome
Source: Front Pediatr. 2025 May 21;13:1553571. doi: 10.3389/fped.2025.1553571 (PMC12134073; doi:10.3389/fped.2025.1553571)
Supplement: Supplementary file 1 [file Datasheet1.pdf]

SUPPLEMENTARY METHODS

Respiratory Syncytial Virus (RSV) Stock preparation

Hep2 cells were seeded at 6 million cells per T-175 flask and allowed to rest overnight. Prior to infection, one flask of Hep2 was counted to confirm expected cell numbers and allow accurate calculation of viral load. RSV-A2 GFP tagged was diluted in serum free EMEM (ATCC) media. Hep2 cells were exposed to 5mL of diluted virus stock for a final concentration of MOI 0.1 for 2 hours with occasional rocking. After 2 hours, 6mL of EMEM supplemented with fetal bovine serum (FBS, ATCC) were added to each flask. Virus was allowed to propagate for 96 hours, or until greater than 50% CPE was seen before harvesting. At time of harvest, supernatant was pooled from all flasks and kept on ice. Cells were covered with 20% sucrose (w/v) in NT buffer and immediately placed at -80°C until the layer froze. Cells were then allowed to thaw gently on ice. Once thawed, cell lysates were scrapped using a cell lifter (VWR) and the lysate was combined with the virus containing supernatant. This crude virus containing solution was then spun at 1000xG for 10 minutes at 4°C to remove cellular debris. The clarified supernatant was then transferred to a new tube. 50% PEG6000 in NT buffer was added to the supernatant for a final concentration of 10% to precipitate virus particles. Rubes were then rocked at 4C for 2 hours, after which, they were centrifuged at 3200xG for 20 minutes at 4°C to pellet virus particles. The resulting pellet was then resuspended in approximately 20x the volume of 20% sucrose in NT buffer. Resuspended virus solution was layered onto a sucrose gradient of 60% and 30% sucrose in NT buffer. Sucrose gradient with virus supernatant tubes were loaded into an SW41Ti rotor and spun at 50,000xG for 1 hour at 4°C. Tubes were then carefully removed from the rotor and a hazy band around the 30/60% sucrose interface was observed. This band was carefully collected and immediately snap frozen in cryovials using a dry ice/ethanol bath. Aliquoted viral stocks were stored at -80°C or below until time of quantification of downstream application.

Molecular studies in AEC cultures

6 well plates were coated with 1mL of PureCol solution (Advanced BioMatrix) diluted 1:30 in sterile H2O. PureCol solution was allowed to incubate in the wells for 1 hour, covered, before being removed. Wells were then washed 3 times with sterile 1x PBS (Gibco). The final wash was removed, and plates were left to dry inside the biosafety cabinet. Once dry, plates were wrapped in foil and stored at 4°C until time of experiment, but no longer than 3 weeks. Human nasal AECs were seeded on PureCol coated plates at 90,000-150,000 cells/well in 1.8mL CRC media.

Poly I:C treatment of human airway epithelial cells

Plated cells were allowed to rest for 24 hours after plating before exposing to a dsDNA viral mimic (Poly I:C) diluted in airway epithelial growth media containing only retinoic acid (RA only media)(PromoCell) to a concentration of 10 ng/µL.

miR155 transfection of human airway epithelial cells

Plated cells were allowed to resto for 24 hours after plating before transfection with 10 nM of miRVana non-targeting control or miR155 mimic (ThermoFisher) and 7µL of Lipofectamine transfection reagent (ThermoFisher) in 300 µL of Opti-MEM media(gibco). Plates were rocked every 30 minutes for 1.5 hours before the total volume of each well was brought up to 1mL with RA only media. 6 hours after the initial transfection, all media was removed from plates and replaced with 1mL of CRC media.

RSV infection of human airway epithelial cells

Cells were allowed to rest for 24 hours post-plating or 72 hours post-transfection before CRC media was replaced with serum free airway epithelial growth media (referred to as Serum Free Media or SF media). SF media is composed of airway epithelial growth media (Promocell). Cells were serum starved for 18-24 hours before introduction of virus. Virus stocks were diluted in RA only media to facilitate minimum growth requirements in conditionally reprogrammed cells. Virus stocks were diluted to an MOI of 1 per well.

Western blotting

At the time of cell harvest, wells were washed with ice-cold 1x PBS, and then 170 µl of RIPA buffer (Pierce, Thermo Fisher) with protease and phosphatase inhibitors (Roche, PhosSTOP, Halt protease inhibitor) was added to each well. Cells were immediately scraped and transferred to an Eppendorf tube. Protein lysates were then transferred to a tube rotator at 4°C for 30 minutes before being stored at -80°C until the time of protein quantification. Protein concentration was determined using a Bradford assay (Pierce, Thermo Fisher).15-30ug of protein were prepared for western blotting, with equal concentrations of protein loaded across the gel. Protein lysates were treated with reducing agent and heated to 95°C for 10 minutes prior to loading on a 4-12% bis-Tris 1.5mm mini gel (Thermo fisher). Protein samples were run using an Invitrogen gel dock at 115v for 1 hour and 45 minutes. Protein was then transferred using the BioRad Turbo Transfer system onto a nitrocellulose membrane. Membranes were blocked for 1 hour in TBST with 5% milk. After blocking, membranes were probed with primary antibody according to manufacturer's protocol. Antibody targets include: BACH1, HO-1, and GAPDH (detailed product information in table provided below). Membranes were probed overnight with primary antibody at 4°C with gentle rocking. The following morning, membranes were probed with corresponding secondary antibodies: antirabbit or anti-mouse (detailed product information provided in the table below). Membranes were incubated with secondary antibody for 1 hour at room temperature with gentle shaking. After incubation, membranes were washed 3 times with TBST and subsequently developed on the BioRad ChemiDoc using either Cytivia Amersham ECL or SuperSignal West Femto Maximum Sensitivity Substrate reagent. Western blot densitometry was performed using the ImageLab Software (BioRad version 5.2.1). All samples were normalized to a-tubulin as a loading control prior to statistical analysis.

Antibody	Source	Identifier	Dilution	Diluent
Anti-BACH1	Santa Cruz	sc-271211	1:1000	5% Milk in TBST
Anti-HO-1	Cell Signaling Technologies	5853S	1:1000	5% BSA in TBST
Anti-GAPDH	Santa Cruz	sc-47724	1:2000	5% Milk in TBST
Goat-Anti-Mouse IgG (H+L) HRP linked secondary (Pierce)	Thermo fisher	31430	1:10000	5% Milk in TBST
Anti-Rabit IgG, HRP-Linked secondary antibody	Cell Signaling Technologies	7074P2	1:2000	5% Milk in TBST

Transcriptome microarrays analysis

A genome-wide transcriptome analysis was performed using the Affymetrix GeneChip Human Clariom D Arrays, following the manufacturer's guidelines (ThermoFisher). Total RNA (100 ng) was used as the starting material for target preparation. The microarrays were then washed, stained, and scanned with the Affymetrix GeneChip Command Console Software, generating .cel files as data output. The array data were analyzed using Transcriptome Analysis Console (TAC) Software 4.0 (ThermoFisher).

RNAseq methods and statistical analyses

RNA sequencing libraries were prepared using the NEBNext Ultra RNA Library Prep Kit from Illumina using the manufacturer's instructions (NEB, Ipswich, MA, USA). RNA was sequenced on Illumina NovaSeq 6000. Quality controls were estimated using fastqc (version 0.11.9) for

individual samples and multiqc for all samples. After quality trimming, the reads were aligned to the human reference (hg38), and the counts estimated with RSEM (version1.3.1). For differential expression, we first filtered out low read counts (< 200 reads) and then estimated sample quality using principal component analyses (PCA). Normalization and differential expression was performed with DESeq2 (version 1.38.3) using an adjusted p-value threshold of <0.05. Functional gene ontology was evaluated using clusterProfiler (version 4.6.2) and reactome database. Hypoxia gene activation was examined with z scores. Genes associated with the pathway term Hypoxia, from the molecular signature database (MSIGDB)[31], were used for the analysis. Of the 200 genes in the database, 50 genes pass the read quality threshold (> 200 reads). Next, we calculated the z-score of each hypoxia gene per sample, and then added up individual gene scores per sample to get the sample z-score, which was used for visualization. To evaluate the functional properties of miR-155, gene targets were identified using miRDB.[25,26] Data were analyzed with the Mann-Whitney U test for group comparisons (e.g., Euploid vs. Trisomy 21) with <0.05 as p-value threshold. All data were analyzed and visualized with the Minitab Statistical Package V.19.1. (Minitab, Inc., State College, PA) and/or R studio (Version: 2023.03.1+446). Raw RNA sequencing data is available in the Gene Expression Omnibus (GEO).
